# The indispensable role of the cerebellum in visual divergent thinking

**DOI:** 10.1038/s41598-020-73679-9

**Published:** 2020-10-06

**Authors:** Zhenni Gao, Xiaojin Liu, Delong Zhang, Ming Liu, Ning Hao

**Affiliations:** 1 School of Psychology and Cognitive Science, East China Normal University, No. 3663, North Zhong Shan Road, Shanghai, 200062 China; 2 Center for the Study of Applied Psychology, Key Laboratory of Mental Health and Cognitive Science of Guangdong Province, School of Psychology, South China Normal University, Guangzhou, China; 3 Institute of Systems Neuroscience, Heinrich Heine University Düsseldorf, Düsseldorf, Germany; 4 Institute of Neuroscience and Medicine (INM-7, Brain and Behaviour), Research Centre Jülich, Jülich, Germany

**Keywords:** Psychology, Cognitive neuroscience

## Abstract

Recent research has shown that the cerebellum is involved not only in motor control but also in higher-level activities, which are closely related to creativity. This study aimed to explore the role of the cerebellum in visual divergent thinking based on its intrinsic activity. To this end, we selected the resting-state fMRI data of high- (*n* = 22) and low-level creativity groups (*n* = 22), and adopted the voxel-wise, seed-wise, and dynamic functional connectivity to identify the differences between the two groups. Furthermore, the topological properties of the cerebello-cerebral network and their relations with visual divergent thinking were calculated. The voxel-wise functional connectivity results indicated group differences across the cerebellar (e.g. lobules VI, VIIb, Crus I, and Crus II) and cerebral regions (e.g. superior frontal cortex, middle frontal cortex, and inferior parietal gyrus), as well as the cerebellar lobules (e.g. lobules VIIIa, IX, and X) and the cerebral brain regions (the cuneus and precentral gyrus). We found a significant correlation between visual divergent thinking and activities of the left lobules VI, VIIb, Crus I, and Crus II, which are associated with executive functions. Our overall results provide novel insight into the important role of the cerebellum in visual divergent thinking.

## Introduction

Since the nineteenth century, it has been widely known that the neurons constitute a stable brain network^1,2^. The network is thought to provide the physiological basis of cognition and behaviour^3,4^. In contrast to task-related functional magnetic resonance imaging (task-fMRI), resting-state functional magnetic resonance imaging (r-fMRI) is a task-free experimental paradigm and resting-state functional connectivity (FC) reflects spontaneous correlative low-frequency blood oxygen level dependent (BOLD) signal fluctuations between separate regions of the human brain^5,6^. Previous studies indicated that most of the cognitive neural activities can be shaped by spontaneous brain activities^7,8^. Therefore, exploring the neural basis of behaviour could be done by exploring the modality of spontaneous activities in the human brain^9^. Creativity is defined as the ability to produce novel, original, and useful products^10,11^, and multiple cognitive processing is involved in the processes of creativity such as generation and evaluation^12^. Visual creative thinking (the production of novel, original, and useful visual form^13^) is an important part of creativity^14^, and visual divergent thinking is a central part of visual creative thinking^10,14^. Visual divergent thinking is an approach to a situation or concept that focuses on exploring as many aspects of the visual concept as possible, and it is a primary component of fields such as photography, drawing, architecture and sculpture^15^. That is, beginning with a single idea, people allow their minds to wander off in numerous directions, gathering multiple thoughts and ideas that are associated with the visual concept. Neuroimaging studies have shown that multiple brain regions are involved in the process of visual divergent thinking, such as the prefrontal cortex (PFC)^15,16^, superior frontal cortex (SFC)^15^, middle frontal cortex (MFC)^17^, and inferior parietal gyrus (IPG)^15^. Meanwhile, visual divergent performance requires information communication among these different brain regions and functional networks that are involved in the default mode network (DMN) (e.g. SFC) and executive function network (EFN) (e.g. MFC and IPG)^15,17^.

A recent published study by Sereno and his colleagues has demonstrated that the surface of the human cerebellar cortex is much more tightly folded than the cerebral cortex, and the human cerebellum constitutes almost 78% of the surface area of the neocortex^18^. This means that, the cerebellum may be involved in highly complex cognitive functions. Concerning the function of the cerebellum, although behaviour and clinical neurology studies have suggested that the cerebellum contributes to movement^19^, motor control^19,20^, and muscular coordination^21^, a growing body of neuroimaging studies have demonstrated that it is also related to cognition and executive functions (e.g. working memory, strategy formation, organising, and cognitive flexibility)^22,23^. Task-fMRI and r-fMRI results have suggested that motor representation is related to the activation of the anterior lobules of the cerebellum and lobule VIII^24^, whereas the posterior lobules of the cerebellum, such as lobules VI–Crus I, lobules Crus II–VIIb, and lobule IX^24^, are thought to be crucial for cognition representation. Lobules VI, VIIb, and Crus I, especially, are involved in the process of executive functions, such as working memory, planning, organizing, and strategy formation, which are important for creative divergent thinking^22,25,26^. Considered together, it is reasonable to speculate that the cerebellum would be involved in visual divergent thinking.

In recent years, although a few creativity studies have indicated that visual divergent thinking is associated with the activities of the cerebellum^27,28^, the role of the cerebellum in visual divergent thinking still unclear. The goal of this study was to extend the body of literature from cerebral brain regions to cerebellar brain regions by answering the following questions: (1) which cerebellar brain regions are associated with visual divergent thinking abilities? (2) Are there any differences in FC between the high-level and low-level creativity groups within the cerebellum? (3) How do the cerebellar regions that are related to visual creative divergent performances interact with the cerebral cortex?

In this study, we explored the neural basis of visual creative divergent thinking based on cerebellar intrinsic activities. We recruited high-level creativity group (HCG) and low-level creativity group (LCG) based on their figural Torrance Test of Creative Thinking (f-TTCT) scores, and then collected the r-fMRI data of each participant in these two groups. We selected 28 cerebellar subregions based on spatially unbiased infra-tentorial (SUIT) template^29^ and calculated the voxel-wise and seed-based FC between these 28 cerebellar sub-regions and cerebral regions for each participant, to trace the connection differences between HCG and LCG. Meanwhile, dynamic measures of the cerebellar network, including dynamic functional connectivity (dFC) and dynamic topological properties, were applied to each participant based on a sliding-window approach. Finally, the topological properties of the cerebello-cerebral network were measured using graph-based analysis.

## Results

### FC within cerebellum

**Figure 1:**
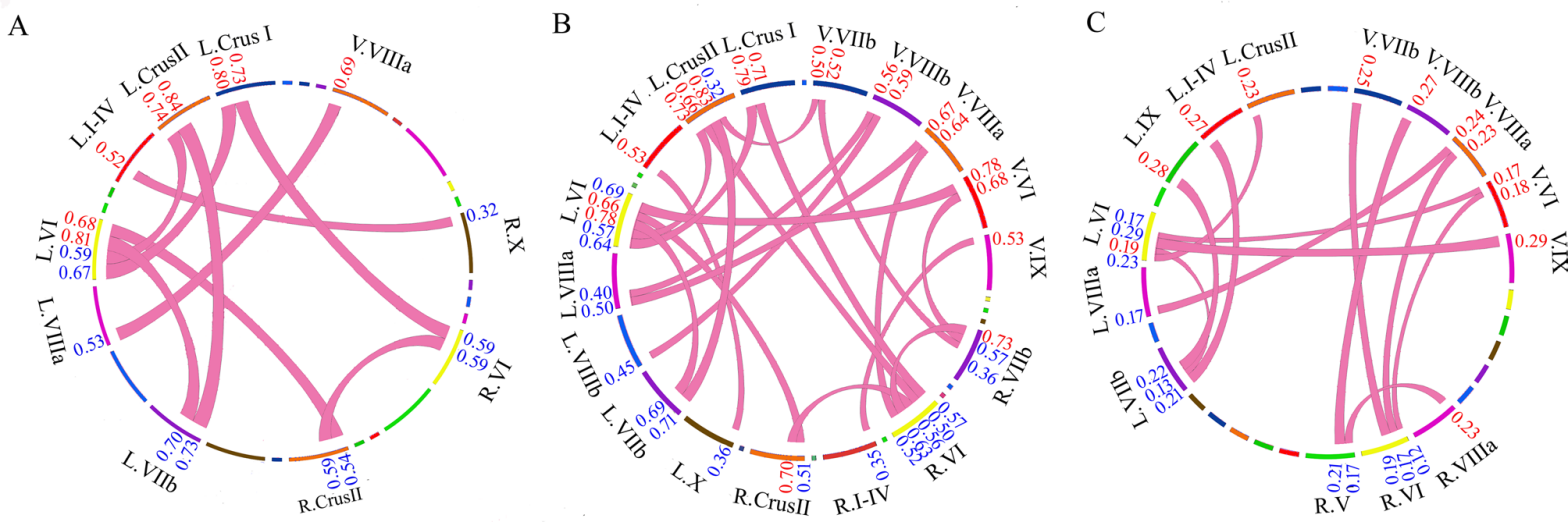
Significant difference (*p* < 0.001) in (**A**) function connectivity (FC), (**B**) averaged dynamic FC (dFC), and (**C**) variability of dFC between high-creativity group (HCG) and low-creativity group (LCG). Note: red numbers, the values of HCG; blue numbers, the values of LCG; L, left hemisphere; R, right hemisphere; V, Vermis.

To test our hypothesis, we selected HCG (*n* = 22) and LCG (*n* = 22) from 180 participants based on f-TTCT scores. We selected 22 participants who obtained the top 12% f-TTCT scores (11 females, 18.86 ± 1.08 years old) as the HCG, and 22 participants with the lowest f-TTCT scores (11 females, 19.13 ± 0.99 years old) as the LCG. We selected a total of 28 cerebellar sub-regions, including 10 cerebellar lobules for each hemisphere (lobules I–IV, V, VI, Crus I, Crus II, VIIb, VIIIa, VIIIb, IX, and X) and eight vermis (vermis VI, Crus I, Crus II, VIIb, VIIIa, VIIIb, IX, and X), to calculate the FC map of each cerebellar subregion using a standard seed-voxel approach for each participant. Significant differences (*p* < 0.001) regarding FC within the cerebellum between HCG and LCG are shown in Fig. 1A and Table 1. To illustrate our results conveniently, we used L. for the left cerebellar lobules, R. for the right cerebellar lobules, and V. for the vermis below. We found significantly higher FC in HCG, mainly within cerebellar lobules compared with LCG. For example, FCs between the L.I–V and R.X, and between the L.VI and bilateral lobule Crus II were significantly higher in HCG than in LCG. Meanwhile, the bilateral Crus I was significantly connected to the bilateral VI, and the L.VIIb was also significantly connected to the L.VI and L.Crus II in HCG compared with LCG. For the vermis region, we found the V.VIIIa was significantly connected to the L.VIIIa in HCG, but LCG was not.

**Table 1:** Significant differences in functional connectivity (FC) within the cerebellum between high- (HCG) and low-level creativity groups (LCG).

FC within cerebellum	HCG	LCG	*p*-value	Effect size (Cohen’s *d*)
L.I-IV–R.X	0.52 ± 0.17	0.32 ± 0.22	0.0008	1.02
L.VI–L.Crus I	0.80 ± 0.07	0.67 ± 0.15	0.0002	1.11
L.VI–L.Crus II	0.74 ± 0.12	0.59 ± 0.16	0.0006	1.06
L.VI–R.Crus II	0.68 ± 0.10	0.54 ± 0.16	0.0002	1.05
L.VI–L.VIIb	0.81 ± 0.07	0.70 ± 0.10	< 0.0001	1.27
R.VI–L.Crus I	0.73 ± 0.11	0.59 ± 0.15	0.0006	1.27
R.VI–R.Crus II	0.71 ± 0.09	0.59 ± 0.15	0.0006	0.97
L.Crus II–L.VIIb	0.84 ± 0.06	0.73 ± 0.14	0.0002	1.02
L.VIIIa–V.VIIIa	0.69 ± 0.15	0.53 ± 0.14	0.0006	1.10

The statistical significance is set at *p* < 0.001. Each FC is given as mean ± SD.

*L* left hemisphere, *R* right hemisphere, *V* vermis.

### dFC and dynamic topological properties of the cerebellar network

Dynamic measures of the cerebellar network including dFC and dynamic topological properties were applied to each participant based on a sliding-window approach^30–32^. We found significant (*p* < 0.001) between-group differences in the averaged dFC of the cerebellar network. Across all sliding-windows, statistical analyses showed significantly higher averaged dFC between the L.VI and five regions in HCG compared with LCG: V.VI, L.Crus I, Crus II, VIIb, and R.Crus II (Fig. 1B, Table 2). The R.VI similarly and significantly connected to five regions, including the V.VIIIa, bilateral Crus II, L. Crus II, and R.VIIb. In addition, the R.VIIb was also significantly connected to the V.VIIb and R.Crus II in HCG compared with LCG.

**Table 2:** Significant differences in averaged dynamic functional connectivity (dFC) within the cerebellum between high-creativity group (HCG) and low-creativity group (LCG).

dFC within cerebellum	Averaged dFC		*p*-value	Effect size (Cohen’s *d*)
	HCG	LCG		
L.I-IV–L.X	0.53 ± 0.16	0.36 ± 0.17	0.0008	1.03
R.I-IV–V.IX	0.53 ± 0.13	0.35 ± 0.20	0.0004	1.07
L.VI–V.VI	0.78 ± 0.07	0.69 ± 0.11	0.0004	0.98
L.VI–L.Crus I	0.79 ± 0.07	0.64 ± 0.17	0.0002	1.15
L.VI–L.Crus II	0.73 ± 0.12	0.57 ± 0.16	0.0004	1.13
L.VI–R.Crus II	0.66 ± 0.10	0.51 ± 0.18	0.0008	1.03
L.VI–L.VIIb	0.78 ± 0.07	0.67 ± 0.10	< 0.0001	1.27
V.VI–R.VIIb	0.68 ± 0.12	0.57 ± 0.12	0.0006	0.92
R.VI–L.Crus I	0.71 ± 0.10	0.57 ± 0.16	0.0006	1.05
R.VI–L.Crus II	0.66 ± 0.14	0.50 ± 0.15	0.0006	1.10
R.VI–R.Crus II	0.70 ± 0.09	0.56 ± 0.15	< 0.0001	1.13
R.VI–R.VIIb	0.73 ± 0.09	0.63 ± 0.12	0.0004	0.94
R.VI–V.VIIIa	0.64 ± 0.12	0.52 ± 0.14	0.0006	0.92
L.Crus II–L.VIIb	0.83 ± 0.06	0.71 ± 0.14	0.0002	1.11
L.Crus II–V.VIIb	0.50 ± 0.18	0.32 ± 0.16	0.0006	1.06
V.VIIb–R.VIIb	0.52 ± 0.16	0.36 ± 0.12	0.0004	1.13
L.VIIIa–V.VIIIa	0.67 ± 0.16	0.50 ± 0.13	< 0.0001	1.17
L.VIIIa–V.VIIIb	0.56 ± 0.18	0.40 ± 0.16	0.0002	0.94
L.VIIIb–V.VIIIb	0.59 ± 0.15	0.45 ± 0.11	0.0006	1.06

The statistical significance is set at *p* < 0.001. Each averaged dFC is given as mean ± SD.

*L* left hemisphere, *R* right hemisphere, *V* vermis.

A significant difference in the variability of dFC across all sliding-windows between HCG and LCG is shown in Fig. 1C. HCG showed a significantly decreased variability of dFC between L.VI and V.VI, V.IX, and L. Crus II and VIIb compared with LCG. Significant between-group differences were similarly found in the variability of dFC between the R.VI and V.VIIb, V.VIIIb, and R.Crus II. Moreover, a decreased variability of dFC between L.VIIb and L.I–IV and L.IX was found in HCG compared with LCG. Table 3 shows more detailed information regarding all significant between-group differences in both the average dFC and its variability.

**Table 3:** Significant differences in variability of dynamic functional connectivity (dFC) within the cerebellum between high-creativity group (HCG) and low-creativity group (LCG).

dFC within cerebellum	Variability of dFC		*p*-value	Effect size (Cohen’s *d*)
	HCG	LCG		
L.I-IV–L.VIIb	0.21 ± 0.06	0.27 ± 0.05	0.0006	1.09
R.V–R.VIIIa	0.17 ± 0.06	0.23 ± 0.06	0.0004	1.00
R.V–V.VIIIb	0.21 ± 0.06	0.27 ± 0.06	0.0006	1.00
L.VI–V.VI	0.12 ± 0.05	0.17 ± 0.06	0.0008	0.91
L.VI–L.Crus II	0.15 ± 0.05	0.23 ± 0.09	0.0004	1.10
L.VI–L.VIIb	0.13 ± 0.04	0.19 ± 0.06	0.0004	1.18
L.VI–V.IX	0.23 ± 0.06	0.29 ± 0.04	0.0002	1.18
V.VI–R.VI	0.12 ± 0.04	0.18 ± 0.05	0.0002	1.33
R.VI–V.VIIb	0.19 ± 0.06	0.26 ± 0.05	0.0004	1.27
R.VI–V.VIIIa	0.17 ± 0.05	0.23 ± 0.07	0.0006	0.99
L.VIIb–L.IX	0.22 ± 0.07	0.28 ± 0.05	0.0006	0.99
L.VIIIa–V.VIIIa	0.17 ± 0.07	0.24 ± 0.06	0.0006	1.07

The statistical significance is set at *p* < 0.001. Each variability of dFC is given as mean ± SD.

*L* left hemisphere, *R* right hemisphere, *V* vermis.

However, we only found significantly (*p* = 0.0020) decreased variability in the characteristic path length of the cerebellar network between HCG and LCG. Table S1 provides all the results of the variability in global parameters for between-group comparison.

### Whole-brain FC map

**Figure 2:**
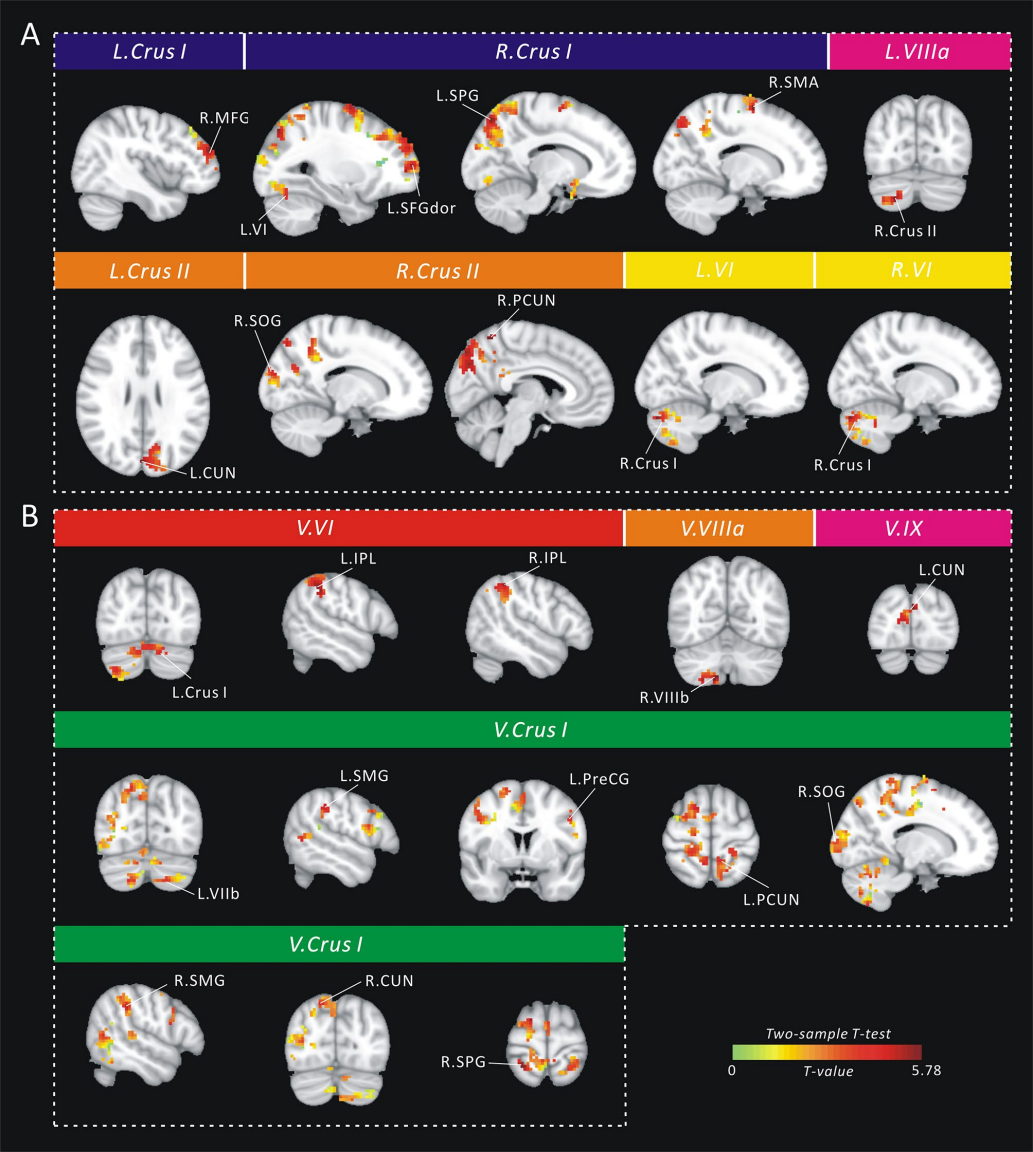
Cluster locations corresponding to the significant between-group difference (*p* < 0.05, Threshold-Free Cluster Enhancement correction, TFCE-correction) in the whole-brain map of (**A**) cerebellar lobules; and (**B**) vermis. *SOG* superior occipital gyrus, *MFG* middle frontal gyrus, *SPG* superior parietal gyrus, *SMA* supplementary motor area, *CUN* cuneus, *PCUN* precuneus, *IPL* inferior parietal lobule, *SMG* supramarginal gyrus, *PreCG* precentral gyrus, *SPG* superior parietal gyrus, *L* left hemisphere, *R* right hemisphere.

We identified each cerebellar subregion with a standard seed-voxel approach for each participant. Significant between-group differences in the whole-brain FC maps of the cerebellar lobules are shown in Fig. 2A and Table S2. Statistical analyses showed that only a significantly (*p* < 0.05, TFCE-correction) higher FC was found in HCG compared with LCG. We found a significantly higher FC between the L.Crus I and right middle frontal gyrus (R.MFG) in HCG compared with LCG. The R.Crus I was significantly connected to four regions, that is, the L.VI, left superior frontal gyrus (dorsolateral) (L.SFGdor), left superior parietal gyrus (L.SPG), and right supplementary motor area (R.SMA). In HCG, the bilateral lobule VI was more significantly connected to the R.Crus I compared with LCG. We also found that the L.Crus II was significantly connected to the left Cuneus (L.CUN), whereas the R.Crus II was significantly connected to the right precuneus (R.PCUN) and R.SOG. In addition, a significantly higher FC between the L.VIIIa and R.Crus II was found in HCG compared with LCG.

Figure 2B and Table S3 show significant (*p* < 0.05, TFCE-correction) differences in the whole-brain FC maps of the vermis between HCG and LCG. The V.VI was more significantly connected to the L.Crus I and bilateral inferior parietal lobule (IPL) in HCG compared with LCG. Meanwhile, we found that the V.Crus I was more significantly connected to eight regions, including the bilateral supramarginal gyrus (SMG), L.VIIb, precentral gyrus (L.PreCG), L.PCUN, R.SOG, R.CUN, and superior parietal gyrus (R.SPG) in HCG compared with LCG. Moreover, significantly higher FCs between the V.VIIIa and R.VIIIb, and between the V.IX and L.CUN were found in HCG and LCG.

### Topological properties of the cerebello-cerebral network

**Figure 3:**
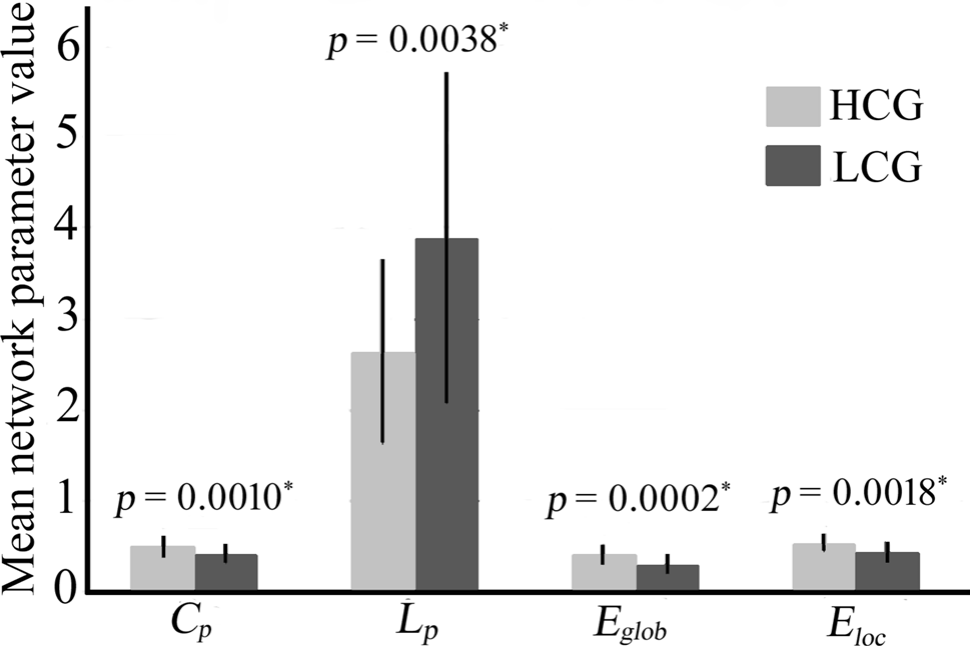
Global parameters of the cerebello-cerebral network for both high-creativity group (HCG) and low-creativity group (LCG). *p < 0.05, Bonferroni correction.

We constructed the cerebello-cerebral network for each participant using a standard seed-wise approach. The topological properties of the cerebello-cerebral network were estimated for each participant based on FC in the cerebellar and cerebral regions, which showed differences between HCG and LCG. Figure 3 and Table S3 present all global parameters of the cerebello-cerebral network for both HCG and LCG. Statistical analyses (*p* < 0.05, Bonferroni correction WE correction) revealed a significantly higher clustering coefficient (*p* = 0.0010), global efficiency (*p* = 0.0002), and local efficiency (*p* = 0.0018) in HCG compared with the LCG. Furthermore, we found a significant between-group difference in characteristic path length (*p* = 0.0038); HCG participants had a shorter characteristic path length compared with LCG.

### Brain–behaviour correlation

We explored a partial correlation (Pearson correlation) between the cerebellar nodal degree and f-TTCT scores. Only those FCs within the cerebellum that showed significant between-group differences were selected to correlate with the f-TTCT scores in this study. We found equally significantly (*p* < 0.05, uncorrected) positive correlations between the values of FC and f-TTCT scores (Figure S1). After Bonferroni correction (*p* < 0.05), we found FCs between the L.VI and L.VIIb tended to be significantly positively correlated with the f-TTCT scores (Figure S1B).

In addition, we found that the f-TTCT scores were significantly positively correlated to the nodal degree of the cerebellar regions in the L.VI, L.VIIb, L.Crus I, and L.Crus II.

## Discussion

This study explored the neural basis of visual divergent thinking from a novel point of view and examined the functional brain connectivity of the inner-cerebellar and cerebello-cerebral regions underlying creative thinking in terms of brain-behaviour correlations and group differences. We summarised the main results as follows. (1) Static/dynamic FC of cerebellar regions indicated group differences across the cerebellar lobules of I–IV, VI, X, VIIb, VIIIa, Crus I and Crus II; (2) information translation efficiency was higher in HCG compared with LCG, along with network stabilisation in the cerebello-cerebral network; (3) the voxel-wise FC results showed group differences across the cerebellar (e.g. lobules VI, VIIb, Crus I, and Crus II) and cerebral regions (e.g. SFC, MFC, and IPG), as well as the cerebellar lobules (e.g. lobules VIIIa, IX, and X) and cerebral brain regions (e.g. the cuneus and precentral gyrus); and (4) the FC between the lobules VI and VIIb showed an obviously positive correlation with visual divergent thinking scores. Meanwhile, the f-TTCT scores and nodal degree of the cerebellar regions showed a significantly positive correlation in the left lobules VI, VIIb, Crus I, and Crus II.

We first compared the differences in FC in the cerebellum between HCG and LCG in a resting state. The results showed the differences in FC between the left lobule VI and bilateral lobule Crus II, the bilateral lobule Crus I and bilateral lobule VI, and the left lobule VIIb and left lobule VI/Crus II in the cerebellum. These between-group differences suggested that high-level creativity individuals may exhibit more efficiency within these cerebello-cerebral brain regions. Creativity is an ability that combines remote concepts and flexibly organises the ideas into creative products^33^, which may be reflected in the resting state^9^. The increased FC strength between these cerebellar regions in HCG suggested that they are crucial for creative thinking. Our brain–behaviour correlation results exhibited a piece of similar evidence. After correction, only the FC between the lobules VI and VIIb was positively related to visual divergent performance scores. Previous studies have shown that the lobules VI and Crus I of the cerebellum contribute to working memory^22,25^, which is an important functional system for the facilitation of a wide range of higher-order cognitive activities such as visual divergent thinking^34^. The lobules VI, VIIb, and Crus I are related to executive functions such as working memory, planning, organising, and strategy formation, which are important for visual divergent thinking^22,26^. The cerebellum has been suggested to be involved not only in movements or motor control but also in attention- and cognition-related activities. The lobules VI, VIIb, and Crus I are involved in various attention- and cognition-related processes. As we know, both visual and verbal creativity requires considerable cognition processes involving working memory^35^, idea generation and selection^36^, solution searching^37^, and insight^38^. The function of the cerebellar lobules VI, VIIb, Crus I, and Crus II support relevant cognitive processes of creativity. High-level creativity individuals are able to deal with questions more precisely and quickly as well as in a novel way, depending on more flexible brain constructions related to creative cognition. Higher FC between these cerebellar brain regions in HCG indicated that higher creativity individuals have more flexible information exchange within the cerebellum. Thus, the higher FC between the cerebellar lobules of VI, VIIb, Crus I, and Crus II in the high-level visual creativity group demonstrated that visual divergent thinking is closely associated with executive function brain regions, including the cerebellar lobules VI, VIIb Crus I and Crus II. Meanwhile, there is a more flexible information exchange in the cerebellar network in high-level visual creative individuals.

Neuroimaging studies have demonstrated the interaction of cerebral regions as well as multiple brain networks during creative divergent thinking^28,39^. In the current study, we used the estimating voxel-wise and ROI-wise FC method to explore the differences in FC between the cerebellar and cerebral regions, including the cognitive and executive network of the cerebellar regions (e.g. lobules VI, VIIb, Crus I, and Crus II), and the cerebral regions of DMN (e.g. SFC) and EFN (e.g. MFC and IPG). Previous fMRI results have demonstrated the crucial regions during visual divergent thinking, including SFC, MFC, and IPG^15–17^. Meanwhile, visual divergent thinking has been argued to be not attributable to cognitive activities in an isolated brain region, but rather to be the results of the interconnection and interaction between many brain regions^15,33,40^ and between different brain cerebral networks^7,27^. Both DMN and EFN are involved in the process of creative divergent thinking^39^. The DMN and EFN regions showed increased coupling during creative divergent thinking processing^39^. Our findings are similar to those in previous studies. The differences were found in creativity-related cerebral network regions (e.g. DMN and EFN) between the two groups. We also found FC between these cerebral networks and cognitive networks of the cerebellum. All these cerebellar and cerebral regions together comprise an interacting creativity-related functional network. The coupling between DMN and EFN helps facilitate novel idea generation^39^. These cerebellar brain regions, which showed differences between the two groups, exhibited a cognitive function that is similar to EFN. Consistently, the co-activation of DMN, EFN, and the cerebellum may reflect both spontaneous and cognitive control of thought, which are both beneficial to visual divergent idea generation and selection.

Similarly, the results of graph-based network analysis provided more evidence that the cerebellum plays an important role in creative thinking. We explored the cerebello-cerebral topological network organisation using path length and network efficiency analyses in this study. Our network organisation results indicated that the cerebello-cerebral network of participants with higher visual divergent creativity exhibited better network efficiency, that is, information translation efficiency was higher in HCG compared with LCG. Meanwhile, our results showed that the average and the variability of shortest path length decreased in HCG compared with LCG; that is, network information translation efficiency was higher in HCG compared with LCG in the cerebello-cerebral network. In total, the cerebello-cerebral network of participants with a high visual divergent creativity level showed better-optimised network organisation compared with that of participants with a low visual divergent creativity level. Our results provide evidence that higher visual divergent creativity individuals have a better cerebello-cerebral network organisation, as well as faster information transmission efficiency. Furthermore, the indicator of cerebellar network variability was smaller in HCG, suggesting that high-level creativity individuals have a more stable cerebellar cognition network, which is more beneficial for visual divergent idea generation.

Intriguingly, we found that the nodal degree of the left cerebellar lobules VI, VIIb, Crus I, and Crus II significantly correlated with f-TTCT scores, which reflect visuospatial creative performance. In the cerebellum, language is heavily right-lateralised, whereas in the cerebral cortex, language is significantly left-lateralised, reflecting crossed cerebello-cerebral projections^22^. A recent fMRI publication by Chen et al. has shown that visuospatial creativity may be characterised by the right hemisphere dominance^41^. Consistent with this, our results showed that visuospatial creative ability may be heavily left-lateralised.

In addition to the above findings, our results showed differences in FC between the left cerebellar lobules of I–IV and right lobule X and between vermis VIIIa and left VIIIa. Meanwhile, the results also showed increased FC between the cerebellar lobules (e.g. lobules VIIIa, IX, and X), which are involved in motor control, and the cerebral sensorimotor regions (e.g. the cuneus and precentral gyrus) in HCG. Previous findings suggest that the sensorimotor cortex and precentral gyrus contribute to visuospatial creative ability, consistent with sensorimotor regions in motor execution, planning and goal-directed behaviour in visual divergent thinking^42,43^. In addition, the relationship between the human body and mind has been generally accepted in the field of cognitive embodied theory. The modern cognitive embodied theory demonstrates that our mental and cognitive processes are embodied in our bodies, depending on our bodies’ specific activity patterns^44^. This means that, cognition is not independent of body movement, but is deeply rooted in the human body and the interaction between the body and our world. Previous evidence has shown that body movement can influence not only inherent concepts, such as attitude, but also the generation of new ideas, which contributes to creativity^45,46^. Slepain and Ambady demonstrated that visual creative thinking can be influenced by certain types of physical movement^46^. Similarly, Oppezzo and Schwartz conducted four experiments, the results of which suggested that walking increased creative ideation in real time and shortly after compared with sitting^47^. Body movement is therefore closely related to creative divergent thinking. Thus, the relation between the sensorimotor cerebellar regions and creative divergent thinking may be crucial in creative divergent thinking.

Although studies in visual divergent thinking have explored the cerebral neural basis, few relevant studies have indicated that visual divergent thinking is associated with the activities of the cerebellum, and the role of the cerebellum in visual divergent thinking is still unclear. Therefore, our findings may provide a different and new insight into the relationship between the cerebellum and visual creative divergent thinking.

Several limitations need to be addressed in further studies. First, we calculated the role of the cerebellum in visual divergent thinking using resting-state data. It is not clear how the cerebellar regions are involved in specific creative thinking tasks and what the interaction is between the cerebellum and cerebral regions in specific creative thinking (i.e. divergent or convergent thinking). Second, we only used visual creative performance scores without verbal creative performances, and it should be determined whether these findings extend to verbal creativity in further studies. Third, these results were from healthy undergraduate students, and it should be determined whether these findings extend to other populations. Finally, we used the SUIT atlas only to extract regions, not to normalise the cerebellum separately. Therefore, the cerebellum is detrimentally affected by whole-brain normalisation.

## Methods

### Participants

We recruited 180 healthy right-handed undergraduates (90 females and 90 males, aged between 18 and 22 years) from South China Normal University. The f-TTCT scores were used as the criterion for selecting participants. The TTCT has shown good predictive validity (*r* > 0.57) and high reliability (*r* > 0.90) for divergent thinking^48,49^. In this study, the f-TTCT was used to measure individual visual divergent thinking performance. The f-TTCT comprises three tasks: picture construction, picture completion, and repeated figures of lines (see details in Figure S2). The scoring of each task comprised four components: originality, flexibility, fluency, and elaboration. The originality scores were assessed using an objective rating method. The originality scores were the number of statistically infrequent ideas and the scoring procedure involved counting the most infrequent responses (i.e. the response was reported by 2% or fewer of the participants in the sample) as ‘2’. If a response was reported by 2–5%, it was scored as ‘1’. All other common responses as ‘0’. Flexibility reflects the ability to shift between conceptual fields. The flexibility scores were the number of different categories of response. Fluency is associated with the ability to generate and consider other possibilities. The fluency scores were based on the total number of relevant ideas that each participant generated. The elaboration shows the subject’s ability to develop and elaborate on ideas. The elaboration scores were assessed using the number of added details of each idea. Our study used the total scores of the f-TTCT to measure the creative thinking of all participants (the sum of originality, flexibility, fluency and elaboration scores). The intellectual ability of each participant was measured using the Combined Raven’s Test (CRT), which has shown high reliability and validity and is recognised widely^50^. We selected the same number of female and male participants to minimise the influence of sex differences. Based on the f-TTCT scores, we selected 44 participants, half of whom obtained the top 12% f-TTCT scores (11 females, 18.86 ± 1.08 years old) as the HCG, while the other half of the participants with the lowest f-TTCT scores (11 females, 19.13 ± 0.99 years old) comprised the LCG. Next, r-fMRI data were collected from these selected participants. The detailed demographic data of the two groups are listed in Table 4. This study was approved by the Institutional Review Board of South China Normal University. All participants gave their informed written consent prior to participation in the current study. All methods used in this study were performed in accordance with the relevant guidelines and regulations of Scientific Report.

**Table 4:** The demographic information of all selected subjects in this study.

	HCG (*n* = 22)	LCG (*n* = 22)	*p* value
Gender (F/M)	11/11	11/11	
Age (years)	18.86 ± 1.08	19.13 ± 0.99	0.388
f-TTCT	65.54 ± 4.09	38.20 ± 5.85	< 0.0001
CRT	54.95 ± 4.85	55.68 ± 3.15	0.558

All *p* values were obtained using the independent sample *t* test.

*HCG* high-creativity group, *LCG* low-creativity group, *f-TTCT* the figural Torrance Test of Creative Thinking, *CRT* Combined Raven's Test.

### MRI data acquisition

All 44 participants were scanned using 3 T Siemens Trio Tim MR scanner at the Brain Imaging Center of South China Normal University, Guangdong, China. The r-fMRI data were collected using a GE-EPI sequence: 32 axial slices; echo time (TE) = 30 ms; repetition time (TR) = 2 s; slice thickness = 3.5 mm; no gap; matrix = 64 × 64; flip angle (FA) = 90°; field of view (FOV) = 192 mm × 192 mm. The participants were instructed to lie down quietly with their eyes closed during the scans.

### Data preprocessing

The r-fMRI data were preprocessed using DPARSF^51^ based on SPM8 (https://www.fil.ion.ucl.ac.uk/spm/software/spm8). The functional preprocessing primarily consisted of the following steps: (1) The first 10 volumes were discarded for signal equilibrium and the remaining 230 images were used in subsequent preprocessing. (2) The time delay of the intra-volume in slices, as well as head movements resulting in geometrical displacements, were corrected (none of the participants were excluded based on the criterion of displacement of > 1 mm or angular rotation of > 1° in any direction). (3) The image data were normalised to the Montreal Neurological Institute (MNI) space at 3-mm isotropic resolution using an echo-planar imaging (EPI) template. (4) The data were band-pass filtered (0.01–0.1 Hz) to decrease the effects of low-frequency drift and high-frequency physiological noise. (5) We removed the linear trend and spatially smoothed the data with an 8-mm FWHW Gaussian kernel. (6) We removed nuisance covariates, including head motion using the Friston 24-parameter model^4,52^, as well as white matter (WM), and cerebrospinal fluid (CSF) signals, using regression. We limited the FC evaluation within the grey matter using the grey matter probability map in the following calculation step.

### Definition of regions of interest (ROIs) in the cerebellum

**Figure 4:**
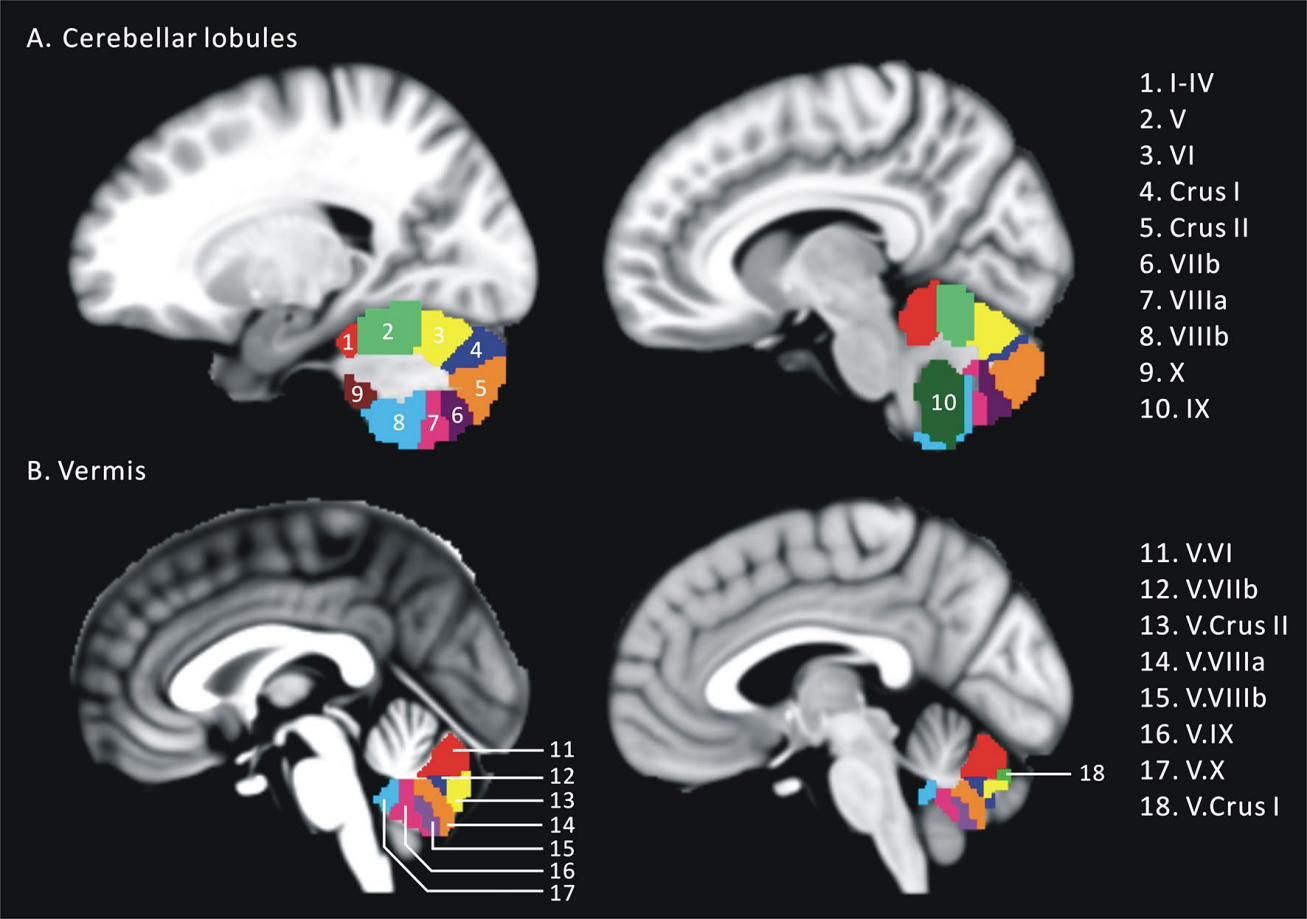
Locations of the cerebellar lobules and vermis (right hemisphere). Each cerebellar regions of interest (ROI) is labeled in different colors with Roman numerals.

We defined the cerebellar ROIs based on the probabilistic MR Atlas of the human cerebellum^53^. This cerebellar atlas was integrally extracted from the Cerebellar toolbox (SUIT, https://figshare.com/articles/Cerebellum_toolbox/1485637). We selected 28 cerebellar subregions, including 10 cerebellar lobules for each hemisphere (lobules I–IV, V, VI, Crus I, Crus II, VIIb, VIIIa, VIIIb, IX, and X) and eight vermis (vermis VI, Crus I, Crus II, VIIb, VIIIa, VIIIb, IX, and X). All cerebellar subregions were resampled to a voxel size of 3 mm^3^ for analysis. Figure 4 shows the different anatomical orientation and slices of these cerebellar subregions.

### FC map of the cerebellar sub-regions

We calculated the FC map of each cerebellar subregion with a standard seed-voxel approach for each participant. Specifically, a given cerebellar sub-region was taken as a seed region, and then, we extracted the averaged time course of all voxels within this seed region for each participant. Using Pearson’s correlation coefficient *r*, we estimated FC between the selected seed regions. After this step, we obtained the FC map of each cerebellar sub-region for each participant. Then, Fisher’s *r*-to-*z* transformation was used to convert these FC maps to *z*-value maps for statistical analysis.

### Dynamic measures of the cerebellar network

Dynamic measures of the cerebellar network including dFC and dynamic topological properties were applied to each participant based on a sliding-window approach^30^ using DynamicBC toolbox^54^. We segmented the time series of all seed regions (cerebellar regions) during a scan into several sliding-windows, and then fixed the length of sliding-windows at 22 TRs (e.g. 44 s) while subsequent sliding-windows would begin with the step of 1 TR. The scanning lasted for about eight minutes, which included 240 TRs; 230 TRs were reserved for the analysis after the pre-processing (10 TRs were removed in the data pre-processing). In total, 209 sliding-windows were generated based on the above calculations for each participant. For each sliding-window, we extracted the time series (22 TRs, 44 s) of each seed region and calculated the Pearson’s correlation coefficient *r* between any two seed regions to identify the dFC of the cerebellar network. Meanwhile, we calculated the dynamic topological properties of the cerebellar network based on the dFC in each sliding-window. Finally, we estimated the average and variability of the dFC and dynamic topological properties across all sliding-windows for each participant and then compared between-group differences.

### Cerebello-cerebral FC map analysis and network construction

We identified each cerebellar subregion with a standard seed-voxel approach for each participant. Specifically, a given cerebellar subregion was taken as a seed region. Then, the averaged time course of all voxels within this seed region and the time course of each voxel in the whole brain were extracted for each participant. We estimated FC, using Pearson’s correlation coefficient *r*, between the selected seed region and each voxel in the whole brain. After this step, Fisher’s *r*-to-*z* transformation was used to convert these FC maps to *z*-value maps for statistical analysis.

We constructed the cerebello-cerebral network for each participant using a standard seed-wise approach. The cerebellar/cerebral regions that showed differences in FC between the two groups were further analysed as the seed regions, including 16 cerebral regions and 11 cerebellar regions. We extracted the averaged time course of all voxels within each seed region and calculated Pearson’s correlation coefficient *r* between any two seed regions to generate the FC within the cerebello-cerebral network. These calculations generated a 27 × 27 connectivity matrix for each participant and were applied in further analysis. Taking all seed region as nodes and FC as edges, we constructed the cerebello-cerebral network for each participant in this study.

### Topological properties of the cerebello-cerebral network

The topological properties of the cerebello-cerebral network were estimated for each participant based on the FC of the cerebellar and cerebral regions, which showed differences between HCG and LCG, using GRETNA^55^ (https://www.nitrc.org/projects/gretna/). To reduce the confounding effects of noisy correlations on network analysis, we only considered the FC that satisfied the threshold of significance-level in this study. Specifically, we assumed that an FC existed only if its corresponding *p*-value met a statistical threshold of *p* < 0.05 (Bonferroni correction) compared with all others in the connectivity matrix. Finally, taking the corrected connectivity matrix, we calculated the topological properties of the cerebellar network including four global parameters: clustering coefficient (*C*_*p*_), characteristic path length (*L*_*p*_), global efficiency (*E*_*glob*_), and local efficiency (*E*_*loc*_). *C*_*p*_ reveals the closeness of the relationship between a node and its neighbouring nodes. *L*_*p*_ characterises the optimal steps for information transfer. *E*_glob_ and *E*_loc_ describe the efficiency of information transfer between different nodes within the cerebello-cerebral network. The definitions and descriptions of these parameters are listed in Table S4.

### Correlation analyses

We explored a partial correlation (Pearson correlation) between the cerebellar nodal degree and f-TTCT scores. We used the False Discovery Rate (FDR) correction^56^ to perform multiple comparison correction. Then, Spearman’s correlation analysis was applied to investigate the correlation between brain measures (e.g. FC and global parameters) and f-TTCT scores. Age and sex were considered as confounding factors and subsequently controlled in all correlation analyses. Correlations with *p* < 0.05 were considered as statistically significant.

### Statistical analyses

A two sample *t*-test was applied to detect the differences in the FC map of the cerebellar subregion and whole-brain regions between HCG and LCG. We determined the clusters, showing statistical between-group differences, using the following criteria: (1) significant threshold *p* < 0.05 with a strict multiple comparison correction strategy, Threshold-Free Cluster Enhancement (TFCE); (2) each cluster should have more than 50 voxels; and (3) the peak voxel of the cluster is located in the grey matter.

We used a nonparametric permutation *t*-test to determine the difference in each FC in the cerebellum and whole-brain regions, topological properties of the cerebello-cerebral network, and the mean and variability of dynamic measures. Age and sex were considered as confounding factors and were thus controlled in these analyses. Briefly, for a given parameter (such as FC, *C*_*p*_, *L*_*p*_, *E*_*glob*_, and *E*_*loc*_), we randomly paired their values between HCG and LCG to generate two new groups. Subsequently, we recalculated the mean value of the two new groups and estimated their differences. This permutation was repeated 5000 times to obtain the empirical distribution of the difference between the two groups. We then selected a significance level of *p* < 0.05 to determine the significant difference between HCG and LCG at 95% of the empirical distribution in a two-tailed test. Given the small sample size of participants in our study, when we found significant between-group differences in any FC, dFC, or global parameter of the cerebellar network, we also calculated for corresponding effect size (Cohen’s *d*) according to Cohen^57^.

### Ethics statements

The ethics protocols were approved by the Institutional Review Board of South China Normal University. All methods used in this study were performed in accordance with the relevant guidelines and regulations of Scientific Report.

## Supplementary information

Supplementary Information.

## Data Availability

All original and processed fMRI images data related to this publication will be available upon request with a legitimate reason.
