# Supplementary material for: The indispensable role of the cerebellum in visual divergent thinking
Source: Sci Rep. 2020 Oct 6;10:16552. doi: 10.1038/s41598-020-73679-9 (PMC7538600; doi:10.1038/s41598-020-73679-9)
Supplement: Supplementary file 1 — Supplementary Information. [file 41598_2020_73679_MOESM1_ESM.pdf]

The indispensable role of the cerebellum in visual divergent thinking

Zhenni Gao[1,a], Xiaojin Liu[3,4,a], Delong Zhang[2], Ming Liu[2], Ning Hao[1*]

[1] School of Psychology and Cognitive Science, East China Normal University, Shanghai, China.

[2] Center for the Study of Applied Psychology, Key Laboratory of Mental Health and Cognitive Science of Guangdong Province, School of Psychology, South China Normal University, Guangzhou, China.

[3] Institute of Systems Neuroscience, Heinrich Heine University Düsseldorf, Düsseldorf, Germany.

[4] Institute of Neuroscience and Medicine (INM-7, Brain and Behaviour), Research Centre Jülich, Jülich, Germany.

[a] These authors contributed equally to this work.

[*] Correspondence author at: School of Psychology and Cognitive Science, East China Normal University, No. 3663, North Zhong Shan Road, Shanghai 200062, China. E-mail address: nhao@psy.ecnu.edu.cn (N. Hao).

Table S1 The variability of global parameters of the cerebellar networks for both high- (HCG) and low-level creativity groups (LCG). Each parameter is given as mean ± SD. * p <0.05 (FWE-corrected).

Global parameters	HCG	LCG	p-value
Clustering coefficient (C_p)	0.08 ± 0.02	0.08 ± 0.02	0.3932
Characteristic path length (L_p)	0.26 ± 0.08	0.34 ± 0.09	0.0020*
Global efficiency (E_{glob})	0.08 ± 0.02	0.09 ± 0.02	0.3186
Local efficiency (E_{loc})	0.07 ± 0.02	0.07 ± 0.01	0.1462

Table S2 Differences in global parameters of the cerebellar networks between high- (HCG) and low-level creativity groups (LCG). The statistical significance is set at p <0.05 (FWE-corrected). Each parameter is given as mean ± SD. *p <0.05 (FWE-corrected).

Global parameters	HCG	LCG	p-value	effect size (cohen's d)
Clustering coefficient (C_p)	0.48 ± 0.09	0.39 ± 0.09	0.0026*	0.90
Characteristic path length (L_p)	2.64 ± 1.02	3.88 ± 1.81	0.0038*	0.53
Global efficiency (E_{glob})	0.41 ± 0.10	0.29 ± 0.09	0.0002*	0.81
Local efficiency (E_{loc})	0.52 ± 0.09	0.41 ± 0.10	0.0018*	0.78

Table S3 Cluster locations and peak coordinates corresponding to the significant difference in whole-brain map of the cerebellar region between high- (HCG) and low-level creativity groups (LCG). The statistical significance is set at p <0.05 (Threshold-Free Cluster Enhancement correction, TFCE-correction).

Seed region (Cerebellar region)	Cluster location	Cluster size (# voxels)	BA	peak coordinate x	y	z	*T* value
Cerebellar lobules							
L. Crus I	R. MFG	251	46	42	51	21	3.9444
R. Crus I	L. VI	122	/	-27	-69	-21	3.5
	L. SFGdor	1365	10	-27	60	9	4.3716
	L. SPG	2087	7	-12	-78	51	4.5472
	R. SMA	281	6	15	-3	66	4.4455
L. VIIIa	R. Crus II	64	/	24	-75	-45	4.0054
L. Crus II	L. CUN	223	3	3	-84	27	3.631
R. Crus II	R. SOG	55	18	15	-93	21	3.4379
	R. PCUN	1079	5	6	-54	60	4.299
L. VI	R. Crus I	593	/	15	-84	-30	5.3343
R. VI	R. Crus I	701	/	15	-84	-30	5.7814
Vermis							
V. VI	L. Crus I	400	/	-12	-78	-30	4.0097
	L. IPL	205	40	-54	-42	42	4.9345
	R. IPL	103	40	51	-42	48	4.7399
V. VIIIa	R. VIIIb	68	/	12	-63	-54	3.8831
V. IX	L. CUN	79	18	0	-87	21	3.6376
V. Crus I	L. VIIb	83	/	-18	-75	-48	3.2567
	L. SMG	164	2	-54	-36	36	3.8617
	L. PreCG	149	44	-45	3	33	3.4767

L. PCUN	191	5	-9	-48	63	3.6461
R. SOG	130	17	15	-102	6	3.0561
R. SMG	86	2	54	-36	36	4.095
R. CUN	133	7	18	-78	48	3.5804
R. SPG	774	7	27	-48	69	4.6842

Abbreviation: SOG, Superior occipital gyrus; MFG, Middle frontal gyrus; SFGdor, Superior frontal gyrus (dorsolateral); SPG, Superior parietal gyrus; SMA, Supplementary motor area; CUN, Cuneus; PCUN, Precuneus; IPL, Inferior parietal lobule; SMG, Supramarginal gyrus; PreCG, Precentral gyrus; SPG, Superior parietal gyrus; BA, Brodmann's area; L, left hemisphere; R, right hemisphere; V, Vermis.

Table S4 Mathematical definitions of the global parameters of a given network *G* (*N*, *M*) with *N* nodes and *M* edges. The detailed descriptions of these network parameters can also be found in Rubinov and Sporns (2010).

Global parameters	Definitions	Descriptions
Clustering coefficient	$C_p = \frac{1}{Ns_i(k_i-1)}\sum_{j,h\in G}\frac{(w_{ij}+w_{ih})}{2}a_{ij}a_{ih}a_{jl}$	The w_{ij} is the weight between node *i* and *j* in a network and s_i is the strength of node *i*. C_p is the mean of the weighted clustering coefficients over all nodes in a network, which indicates the extent of local interconnectivity or cliquishness in a network.
Characteristic path length	$L_p = \frac{1}{N(N-1)}\sum_{i=1}^{N}\sum_{j=1,j\neq i}^{N} L_{ij}$	*N* is the total number of nodes. L_{ij} is the characteristic path length between nodes *i* and *j*. L_w measures a harmonic mean length between all pairs of nodes and quantifies the ability for information propagation in parallel over the whole network.
Global efficiency	$E_{glob} = 1/L_p$	E_{glob} is the inverse of the harmonic mean of the characteristic path length between each pair of nodes within the network. E_{glob} measures the global efficiency of parallel information transfer in the network.
Local Efficiency	$E_{loc} = \frac{1}{N}\sum_{i=1}^{N} E_{glob}(G_i)$	E_{glob} (G_i) indicates the global efficiency of the subgraph G_i composed of the neighbors of node *i*. E_{loc} is the average of the local efficiencies across all nodes. It reflects how the communication efficient changes among the neighbors of node *i* when node *i* is removed.

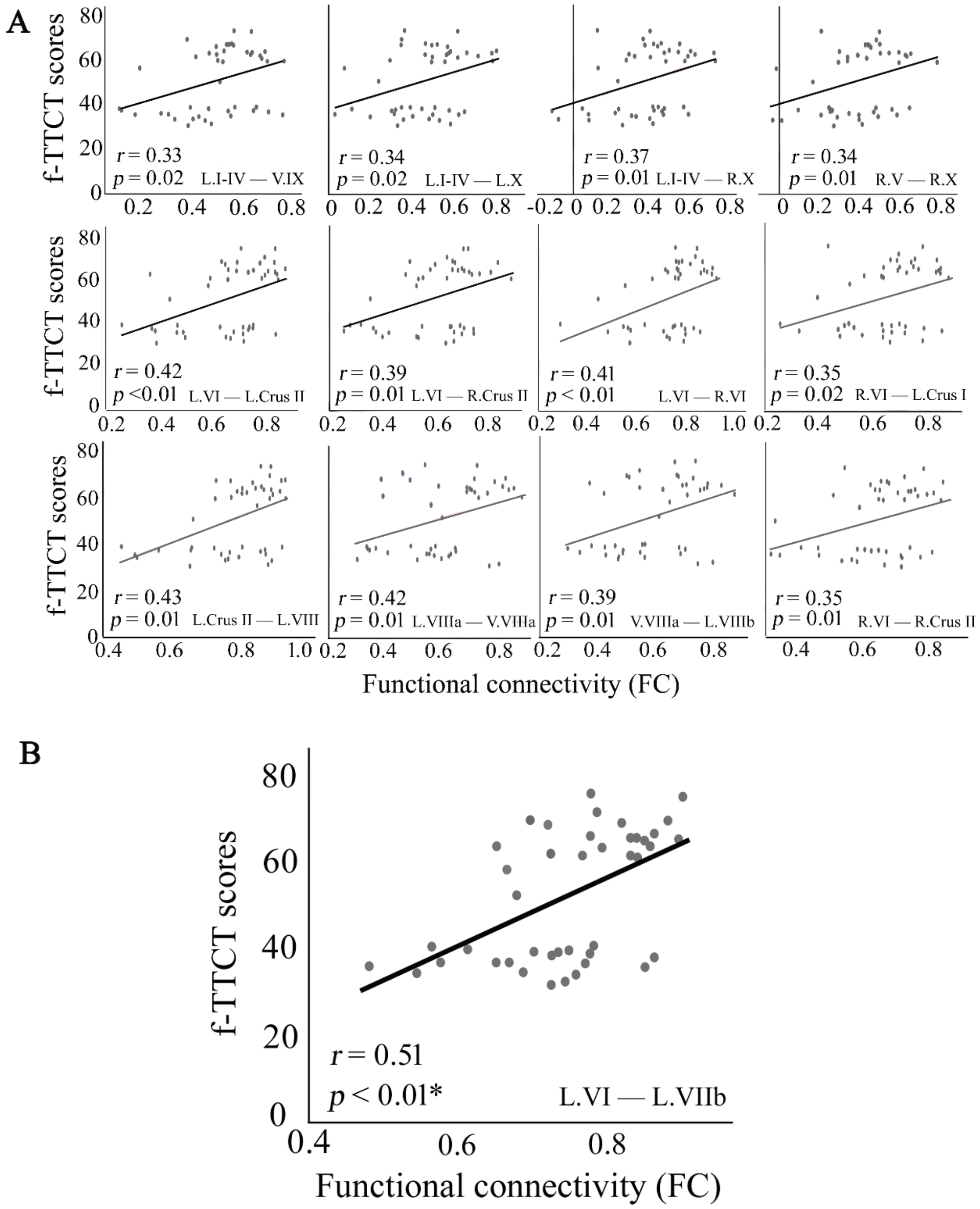

Figure S1 Correlations between the functional connectivity (FC) and figural Torrance Test of Creative Thinking scores (f-TTCT scores). A) Both significant between-group differences in FC significantly (p <0.05, uncorrected) positively correlated with the f-TTCT scores. B) After the family-wise error correction (FWE-correction) (p < 0.05), only the FC between the L.VI and L.VIIb significantly negatively correlated with the f-TTCT scores. * p <0.05, FWE-correction.

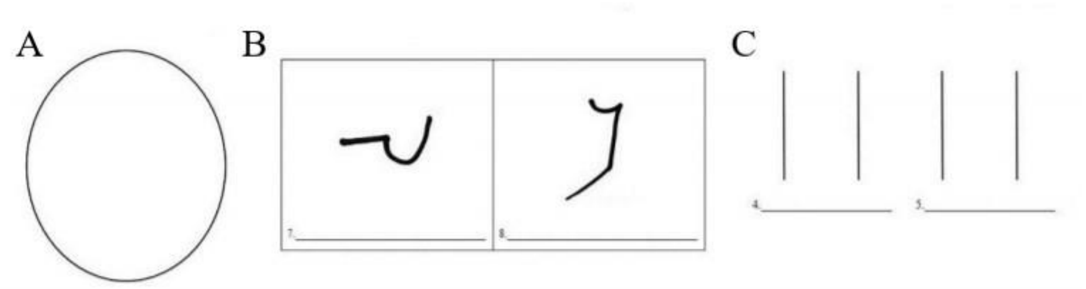

Figure S2 Samples of the figural Torrance Test of Creative Thinking. A) picture construction, B) picture completion, and C) repeated figures of lines. In picture construction task, the subjects were asked to construct a creative picture that told a story based on a circle. In picture completion part, the subjects were required to complete 10 different lines to novel and interesting pictures. In repeated figures of lines part, subjects were asked to construct novel and meaningful pictures based on 10 pairs of parallel lines. Each part should be finished in ten minutes.
